# Does psychiatry need religion and spirituality in its treatment approach? Narcissism as an example

**DOI:** 10.4102/sajpsychiatry.v22i1.563

**Published:** 2016-06-10

**Authors:** Manfred Böhmer

**Affiliations:** 1 Department of Psychiatry, University of Pretoria, South Africa

## Abstract

**Background:**

Although religion and spirituality are increasingly recognised as important in the understanding and treatment of patients, there are also concerns about their role in psychiatry. The recommendation for the integration of spirituality in the approach to psychiatric practice highlights the importance to further think about this practice.

**Objective:**

To contribute to the debate on the role of spirituality in psychiatry by considering the opinions of two prominent thinkers in this field, the theologian Tillich, and the psychoanalyst Symington.

**Method:**

The approach of Tillich and Symington to mental health problems are compared. Narcissism is focussed on, since Symington describes narcissism as the core of all pathology and states that the prime aim of psychoanalysis is the transformation of narcissism. The contributions of Kohut and Kernberg are also briefly discussed.

**Results:**

In Symington’s opinion more than psychoanalysis is needed to help those in the grip of narcissism. Tillich emphasises the difference between existential anxiety and anxiety due to psychopathology. Psychotherapy can only heal the latter. Yet he also states that we are incapable of change without self-acceptance. For this a larger experience of acceptance or grace is needed, since we are incapable of offering ourselves this type of acceptance.

**Conclusion:**

In the struggle to get a grip on narcissism, good nurturing experiences, transformative selfobjects, a confrontation with the darker sides of the self and the message of ultimate acceptance are needed. Religion and spirituality have an important contribution to make to psychiatric or psychotherapeutic treatment.

Religion and spirituality are increasingly recognised as important in the understanding of psychiatric disorders and in the clinical assessment and treatment of patients.^1,2,3,4,5^ Yet there are also concerns about the role of religion and spirituality in psychiatry. Curlin et al. report that 82% of psychiatrists are of opinion that religion and spirituality can cause increased suffering, for example, guilt or anxiety.^3^ The suffering of vulnerable people under their harsh beliefs and understanding of God is one of many examples where religion has done a lot of harm.^6^

Religious faith can, however, also enhance well-being.^7^ It is associated with a higher sense of personal empowerment and can provide patients with a positive sense of self and can give meaning to their illnesses.^8,9^

Religion initially referred to something dynamic. It was conceptualised as a verb, stemming from the Latin ‘re-ligare’, to re-connect,^10^ or ‘that which connects’ (p. 14).^11^ Nowadays religion refers to the dogmatic contents of faith, in theistic religions the question of *what* one believes about God.^12^ Spirituality is defined as the search for the sacred,^13^ a reaching out to something beyond ourselves.^14^

The use of the word spirituality as a term and construct in scientific discussions is relatively new.^11^ In this context, spirituality is described in a positive, dynamic and personal way; it is growth-orientated,^11^ a motivating force and ‘has come to represent individuals’ efforts at reaching a variety of sacred or existential goals in life, such as finding meaning, wholeness, inner potential and interconnections with others’ (p. 24).^13^

The recommendation for the integration of spirituality in the approach to psychiatric practice^2^ emphasises the importance to further think about the role of spirituality and religion in psychiatry. Is religion merely a passive way of coping or is it a vital resource?^7^ And does an integrated approach to treatment that includes spirituality and religion enhance the effects of standard psychiatric care?^7^

The aim of this paper is to address these questions by asking whether psychotherapeutic help is sufficient in helping someone suffering from pathological narcissism, or whether religious and spiritual help can be of value or is needed. To pursue this question, the focus of this article is mostly on some of the ideas of the psychoanalyst Neville Symington and the theologian Paul Tillich.

## Narcissism

The term ‘narcissism’ originates from the Greek myth of Narcissus, the beautiful youth who fell in love with an image of himself. Although Freud’s landmark essay *On Narcissism: An Introduction* appeared in 1914,^15^ the term and concept became more widespread in the 1970s, due in great part to the work of the psychoanalysts Kernberg and Kohut.^16^

Narcissism is a complex phenomenon and difficult to define because of the multiplicity of meanings it has acquired. In its colloquial usage, it is used to refer to self-centred persons, often in a pejorative way, but it also refers to a pathological clinical syndrome.^17^ For some, narcissism is synonymous with self-esteem, an indication of the strength of the self.^18^ According to Val, this causes confusion because self-esteem refers to an experiential state and narcissism to a functional concept.^18^ The distinction between healthy and pathological degrees of narcissism is, according to Gabbard, fraught with difficulty.^19^ A certain measure of self-love is normal and desirable. Hence, Kohut boldly defined narcissism as a desirable and even healthy dimension of mature selfhood.^16^

To complicate things, we live in a narcissistic culture,^19,20^ in which ‘being number one is the most important goal of all’ (p. 484).^19^ ‘Making it’ has become more important than promoting values such as commitment, loyalty, integrity and interpersonal warmth (p. 484).^19^ Interpersonal exploitiveness is highly adaptive in our society and winning forgives everything.

Freud, in his introduction into the problem of narcissism, grappled with the problem of the relationship between the ego and external objects or people, leading him to distinguish between ego-libido (libido directed inwards) and object-libido (libido directed outwards).^15^ Narcissism, according to him, means that libido is withdrawn from the external world and instead invested in the ego; it is directed inwards.

In later developments, the work of Kohut and Kernberg helped to coin the concept of a narcissistic personality disorder,^16^ defined in DSM-5 as people showing a pervasive pattern of grandiosity, need for admiration and lack of empathy.^21^ Their work also demonstrates something of the continuum of narcissistic personality disorder – Kernberg described an envious, greedy type, whereas Kohut described a vulnerable type, prone to self-fragmentation.^19^ According to Kernberg, at the deepest level the self-concept of the narcissist is one ‘of a hungry, enraged, empty self, full of impotent anger at being frustrated, and fearful of a world which seems as hateful and revengeful as the patient himself’ (p. 57).^22^ Kernberg is of the opinion that self-hatred rather than self-love lies at the root of pathological narcissism.^17^ Narcissism is linked to feelings of inferiority and the inability to accept oneself.

Kohut sees a weakened or defective self at the centre of a narcissistic personality disorder.^23^ Faulty interaction between the child and his self-objects leads to a damaged self. (The term self-object, coined by Kohut, refers to an object experienced as part of the self. He described two types – the mirroring self-object, representing the need for affirmation, and the idealised parent imago, representing the need for being like the parent or later like the therapist.)^19,23^ Nurturing self-object relationships with parents or significant others helps the child develop a healthy self-esteem.

For Kohut, empathy was the cornerstone of psychotherapy or psychoanalysis.^24^ The goal was to help the patient identify and seek out appropriate self-objects.^19^ Initially, the therapist would function as a positive self-object in relation to whom the person could resume narcissistic development arrested in childhood.^25^

### Symington’s view of spirituality and narcissism

Symington, a psychoanalyst who initially studied theology and philosophy, writes that narcissism is ‘the core of all pathology’ (p. 2) and the source of numerous problems.^26^ For him, narcissism always refers to something pathological and destructive. Healthy self-confidence, he writes, is something different.

Symington describes narcissism as a constellation consisting of a pattern of interlocking elements.^26,27^ The inner state of the narcissist is a brittle and fragmented one, hidden by an imposing crust conveying a sense of apparent strength. Various forces drive such a person as he is lacking an autonomous centre. Envy, greed, jealousy, dependency, paranoia or a primitive hatred, and destructiveness create inner havoc, and an attitude of omnipotence covers up feelings of worthlessness. As the ego is taken as the love object, relationships are one-sided and shallow with little empathy for the feelings of others.^26,28^

According to Symington, the prime aim of psychoanalysis is to transform narcissism.^26,28^ Psychoanalytic thinking suggests that parts of the personality can be split off. These unconscious, unintegrated parts can be destructive, for example, intense envy or paranoia projected onto others, leading to interpersonal difficulties. To become conscious of this and to integrate such parts into the personality, to own what belongs to oneself, is described by Symington as a virtuous act.^28^ In psychoanalysis, through interpretation, the patient can come to know himself and begin this process of integration, of constructing his inner and outer world anew.^28^ By using the word ‘virtuous’, Symington already hints that for him psychoanalysis is a spiritual endeavour.

Symington suggests that primitive religion is concerned with survival, with external and placatory acts towards a god, to please the one in power, and this primitive mental attitude still exists in all forms of religious life.^28^ In contrast to this, mature religion teaches that life has a deeper meaning, which transcends death and aims at the transformation of mind and heart. Central to Symington’s thoughts is the idea of ‘natural religion’:^28^ religious truth can be established through reason and without any revelation, the goal is ‘the good’ and the existence of God is not the central element (pp. 42, 171). Natural religion so described is a mature religion and the only religion capable of infusing the emotional life of human beings.^28^

A spiritual person is described by Symington as someone who gives priority to the spiritual quest to discover the intentional basis of his actions.^28^ The goal is to detach oneself from those actions that are bad and to pursue those that are good, to purify one’s intentions and to make the good an internal possession (p. 172). The motivation behind this is self-transcendence, the realisation that life is not only about me. He describes mystics as men devoted to an inner scrutiny with the goal of triumphing over the bad and establishing the good:^28^

The internal possession of the good is what guided these mystics, who were also founders of the great religious traditions. They all founded institutions, which embodied the good in a scriptural canon … This marks the transition from spirituality to religion. A religion, then, is an institution whose goal is the good … in mature religion it [*the good*] is the salvation of the individual members through meaning. (p. 171)

For Symington, mature religion and psychoanalysis have a common spiritual goal: the transformation of destructive action into constructive action.^28^ ‘However, the spiritual domain of religious people has been conscious, whereas the endeavour of those engaged in psychoanalysis is to tackle a sphere of mind which is unconscious’ (p. 182).^28^ In this enterprise, traditional religion is for Symington no longer relevant because it does not have knowledge about these aspects of the psyche and of the sphere of emotional action that exists between people who live in intimacy with one another. Furthermore, traditional religion does not know how to approach problems stemming from this. It does not have the necessary understanding to approach a problem such as narcissism.

In narcissism, the ego takes its own self as a love object.^28^ This unconscious act, writes Symington, implies having a choice and with it the negation of a different possibility. It is this negated possibility that, according to Symington, lies at the heart of narcissism. To describe the mental object that is negated in narcissism but chosen by the healthy minded he coins the term ‘Lifegiver’.^28^ In describing the term ‘Lifegiver’, he writes that in the crises of life the mentally healthy person:

relies on something within through which he or she will cooperate with others. I propose to call this inner quality the Lifegiver … It is a mental object which only comes into existence in the act of being chosen … as, for example, friendship only comes into being in the act of being forged … The act through which the Lifegiver is chosen brings about this mental reality as an inner possession. The person has then the inner equipment necessary for the crises of life … The Lifegiver is an object to which he has a relation if he has made [*a*] fundamental ‘Yes’ [*to life*]. (pp. 122–124)

At the heart of narcissism, according to Symington, lies the basic refusal of the ‘Lifegiver’, a ‘No’, a negativity towards life and towards the other.^28^ He states that an important consequence of this refusal is unconscious guilt, an inner experience of feeling bad, and needing constant approval from others. For Symington, there is thus no such thing as positive narcissism; negativity is an inherent part of the narcissistic structure.

In summary, Symington defines psychoanalysis as a mature natural religion, a mental discipline, with the goal of purifying one’s intentions and motivations. He shares Fromm’s opinion that humanity can do this without help from any greater-than-human power.^29^ It is within the power of a person to choose and to make ‘the good’ part of himself.^28^

## Tillich: Integration of religion and psychology

Paul Tillich (1886–1965) served as a military chaplain in World War I. This shattering experience was for him a stark reminder that autonomy as a sole guide for human action was indeed questionable.^30^ He was an early critic of Hitler and the Nazi movement, and in retaliation he was barred from German universities in 1933. He then accepted an invitation to join the faculty at Union Theological Seminary in New York.^30^ Tillich, who was deeply rooted in Lutheran theology, has been described as the greatest twentieth-century theological representative of the psychology and theology dialogue.^29^ He played a leading role in the New York Psychology Group, which met regularly from 1941 to 1945 and included prominent psychotherapists such as Erich Fromm, Rollo May and Carl Rogers.^29^

Tillich sees man as estranged and alienated from his essential being. This separation is threefold: separation of man from himself, separation amongst individual lives and separation of all men from the Ground of Being. Sin, for Tillich,^31^ does not mean an immoral act; sin rather refers to this estranged state of being. *Sin is separation* (p. 154).^31^ Sin is the universal and tragic estrangement and alienation of man from his essential being, from that to which he really belongs; sin is the ‘fall’ from essence into existence. *Existence is separation!* (p. 155).^31^

This estrangement from the Ground of Being and from ourselves leads to aggression, hate and despair.^31^ It leads to selfishness and self-hate, which Tillich sees as our real enemy. He joins Jung, who stated that no one can boast that he has fully accepted himself.^29,32^ Feelings of inadequacy associated with self-preoccupation are the problem. Only a person who has overcome self-contempt and learned to love him- or herself will be able to love and to overcome contempt for others.^31^

In discussing the link between psychoanalysis (or psychotherapy) and theology, Tillich is of the opinion that psychoanalysis has much to offer theology and that psychoanalysis and theology need not be seen as being in opposition to each other but rather as being intimately interwoven. Psychoanalysis is about the existential situation of man and the theology about the relationship between this existential situation and the essential nature of man.^29,33^

Tillich writes that psychoanalysis and existentialism have the common goal of describing the existential position of man, an existence of existential alienation, estrangement and finiteness.^33,34^ Existentialism and psychoanalysis furthermore share common roots based in the protest against the growing influence of the philosophy of consciousness. In opposition to this, existentialism and psychoanalysis see man as being driven by unconscious impulses and irrational urges. Existentialism, however, describes a situation of existential anxiety and alienation valid for everyone, whereas psychoanalysis explores the different ways in which man tries to escape his existential problems ‘by fleeing into neurosis and falling into psychosis’ (p. 118).^33^ Being neurotic means not being able to bear with reality as it really is.^35^ According to Tillich,^35^ mental illness implies that a ‘centered being, a person as a centered being, disintegrates’(p. 142). ‘They [*the mental illnesses*] are all related to the center. … This can be healed’ (p. 142). Tillich implies here that healing through psychotherapy or psychoanalysis is about the re-establishment of a centre, it can lead to the re-integration of a person.

Yet, he also says that our real enemy, selfishness or self-hate, cannot be overcome through a Pelagian attempt to muster self-love (Pelagius, 354–418 AD, emphasised the essential goodness of human nature and the freedom of the human will).^36^ Tillich described Fromm’s belief that humanity can overcome its problems without any outside help as lacking depth and insight about the irrational side of man. Man is too alienated, estranged and sick to be able to accomplish this on his own.^29^ A larger experience of acceptance is necessary before self-acceptance and self-love is possible. This larger experience is one of grace:^31^

In grace something is overcome; grace occurs ‘in spite of’ something; grace occurs in spite of separation and estrangement. Grace is the *re*union of life with life, the *re*conciliation of the self with itself. Grace is the acceptance of that which is rejected. (p. 156)

Grace is however not the willingness of a divine father to forgive the weakness of his children. Such a belief needs to be rejected as it is a childish destruction of human dignity. It is thus not about *sins* in a moral sense but about overcoming separation, which, as mentioned, is how Tillich defines sin. This separation is overcome through acceptance: ‘*You are accepted*, accepted by that which is greater than you…’ (p. 162).^31^ Grace means unconditional acceptance. The courage to be is the courage to accept oneself as accepted in spite of being unacceptable, in spite of existential guilt.^37^ This is the basis for the courage of confidence. Only this larger experience of acceptance, and the belief in it, can lead to self-acceptance, and only acceptance, never commands, can lead to transformation.

Self-acceptance is only possible if one is accepted in a person-to-person relation.^37^ The objective power of acceptance is always embodied in a person, here the therapist, who can recognise guilt, who can judge and who can accept in spite of the judgment. Tillich writes that the psychotherapist should not have spiritual counselling as prime focus, but that in the Christian tradition of Protestantism everyone can be a priest to the other.^38^ In this situation, the psychotherapist does not act in a psychotherapeutic sense but ‘appeals to the center of the personality to actualize an ultimate concern’ (p. 142).^35^ Healing of the whole person not only involves bodily, psychological and sociological healing but also spiritual healing, that is, addressing the matter of ultimate concern, which is finding ‘the ultimate center in which our center can rest’ (p. 143).^35^

## Treatment of narcissism

The psychoanalysts Kernberg and Kohut represent two contrasting approaches to the understanding and treatment of narcissism. For Kohut, narcissism is the result of the parent’s empathic failures, parents not responding with enough validation and admiration to satisfy the child’s needs.^19^ In his treatment of such patients, empathy was a cornerstone and acceptance by the therapist of vital importance. Kohut believed that the therapeutic relationship and the internalisation of the other as a transformative self-object could facilitate the resumption of healthy narcissistic development arrested in childhood.^25^ Kernberg’s approach is in general more confrontational.^19^ Interpretation and confrontation, especially of idealisation, greed and envy, play an important role. For Kernberg, it is important that the patient recognises and develops an understanding of his child’s contribution to his problems, and that the patient integrates idealisation and trust with rage and contempt, and develops concern for others.^19^

Symington extends the boundaries of psychoanalysis; for him, it becomes a spiritual quest. Self-transcendence means to curb narcissism, to internalise ‘the good’ and to establish constructive relations. Reason is the tool needed to define ‘the good’. This psychoanalytic struggle is an act of virtue.^28^ In this struggle, the bits and pieces of a disintegrated individual can be integrated into an organised unity with as result the formation of a person.^26,27^

It is interesting how primitive hatred and self-hate, linked to a lack of love and self-love, are seen as central problems of narcissism and emotional problems by Kernberg, Symington and Tillich.

### Tillich and Symington: similarities, inconsistencies and difficulties

The relationship between psychoanalysis and religion is important for both Tillich and Symington. Both state that psychoanalysis and religion can heal and help overcome a state of disintegration.^28,35^

Both are not without contradictions. Symington speaks of psychoanalysis as a virtuous undertaking and a treatment for narcissism,^28^ yet in a later book he writes that analysis alone was not enough to heal him, nor the majority of analysts and therapists, from narcissism.^26^ That was something, he writes,^26^ he had to do for himself:

with the aid of many different teachers, thinkers and disciplines of thought. … To be sane, it is necessary to ‘stand outside of oneself’, and this can never be done within one discipline of thought or ideology. (p. 12)

According to him, it is extremely difficult to reduce the power of narcissism in the personality and a person would be extremely lucky if he achieved this outcome. Contributing to this, he writes,^26^ ’is that the majority of psychoanalysts and psychotherapists are themselves very narcissistic’ (p. 3). If true, this would be a very sobering statement, considering that psychoanalysts have to undergo four to five times-a-week training analysis for several years.

Narcissism as such is not addressed by Tillich. He, however, describes our real problems as being self-hate, self-contempt and selfishness, as well as feeling insecure and inadequate and not able to fully accept oneself. His description of psychopathology is very similar to the psychodynamic understanding of the narcissistic structure.

In Tillich’s opinion, psychoanalysis or psychotherapy can help with neurotic problems, with general psychopathology, but that it cannot deal with the ultimate existential issues of finitude, guilt and emptiness.^37,38^ Yet he also states that we are incapable of change without self-acceptance. A larger experience of acceptance or grace is needed before self-acceptance is possible.^29^

Both Symington and Tillich thus note that something more than just psychoanalysis or psychotherapy is needed. The difference, however, is that Symington writes that he had to do it *for himself*, whilst Tillich says that grace, something *bestowed upon us*, is needed. For Tillich, man is not a virtuous creature, virtue is not something we can acquire. Only God is ‘the good’. Virtuous behaviour can only be an outflow of grace (the question of why grace is bestowed upon some and not others, bringing up the complicated problem of theodicy, falls outside the scope of this article).

Kohut, Kernberg and Symington have made valuable contributions to the understanding of narcissism and their approaches need to be integrated in the treatment of narcissism. But narcissism is a very difficult condition to treat and a purely psychoanalytic approach might not be enough for most patients. The question remains what this ‘more’ is that is needed. For Tillich, this is the realisation of acceptance by the Ground of Being, who becomes, using Symington’s concept, our Lifegiver. This can only be experienced through an accepting therapist who points towards a source of acceptance, which transcends both therapist and patient.^29^

## Concluding remarks

The aim of this article is not to promote a specific religion but rather to think about the borders of psychotherapy, religion and spirituality. This paper also suggests that the basic, unspoken existential stance of the therapist and his or her thinking about the essence of being will silently speak in the therapeutic process.

Narcissism, the result of early, severe deprivation and a lack of secure attachment, is an ever-present and pervasive problem – a defence against man’s underlying anxiety and fragility. In the struggle to get a grip on this, good nurturing experiences, transformative self-objects, a confrontation with the darker sides of the self and the message of ultimate acceptance or grace are all needed. Religion and spirituality thus have an important contribution to make to psychiatric or psychotherapeutic treatment:^31^

We cannot compel anyone to accept himself. But sometimes it happens that we receive the power to say ‘yes’ to ourselves, that peace enters into us and makes us whole … Then we can say that grace has come upon us. (p. 163)
